# House mouse colonization patterns on the sub-Antarctic Kerguelen Archipelago suggest singular primary invasions and resilience against re-invasion

**DOI:** 10.1186/1471-2148-10-325

**Published:** 2010-10-26

**Authors:** Emilie A Hardouin, Jean-Louis Chapuis, Mark I Stevens, Jansen Bettine van Vuuren, Petra Quillfeldt, Rick J Scavetta, Meike Teschke, Diethard Tautz

**Affiliations:** 1 Max Planck Institute of Evolutionary Biology, August-Thienemann-Str. 2, 24306 Plön, Germany; 2 National Museum of Natural History, UMR 7204, Conservation des espèces, restauration et suivi des populations, 61 rue Buffon, CP n°53, 75005 Paris, France; 3 South Australian Museum, North Terrace, Adelaide SA 5000, Australia; 4 University of Adelaide, School of Earth and Environmental Sciences, Adelaide SA 5000, Australia; 5 Centre for Invasion Biology, Stellenbosch University, Private Bag X1, Matieland 7602, South Africa; 6 Max Planck Institute for Ornithology, Vogelwarte Radolfzell, Schlossallee 2, D-78315 Radolfzell, Germany

## Abstract

**Background:**

Starting from Western Europe, the house mouse (*Mus musculus domesticus*) has spread across the globe in historic times. However, most oceanic islands were colonized by mice only within the past 300 years. This makes them an excellent model for studying the evolutionary processes during early stages of new colonization. We have focused here on the Kerguelen Archipelago, located within the sub-Antarctic area and compare the patterns with samples from other Southern Ocean islands.

**Results:**

We have typed 18 autosomal and six Y-chromosomal microsatellite loci and obtained mitochondrial D-loop sequences for a total of 534 samples, mainly from the Kerguelen Archipelago, but also from the Falkland Islands, Marion Island, Amsterdam Island, Antipodes Island, Macquarie Island, Auckland Islands and one sample from South Georgia. We find that most of the mice on the Kerguelen Archipelago have the same mitochondrial haplotype and all share the same major Y-chromosomal haplotype. Two small islands (Cochons Island and Cimetière Island) within the archipelago show a different mitochondrial haplotype, are genetically distinct for autosomal loci, but share the major Y-chromosomal haplotype. In the mitochondrial D-loop sequences, we find several single step mutational derivatives of one of the major mitochondrial haplotypes, suggesting an unusually high mutation rate, or the occurrence of selective sweeps in mitochondria.

**Conclusions:**

Although there was heavy ship traffic for over a hundred years to the Kerguelen Archipelago, it appears that the mice that have arrived first have colonized the main island (Grande Terre) and most of the associated small islands. The second invasion that we see in our data has occurred on islands that are detached from Grande Terre and were likely to have had no resident mice prior to their arrival. The genetic data suggest that the mice of both primary invasions originated from related source populations. Our data suggest that an area colonized by mice is refractory to further introgression, possibly due to fast adaptations of the resident mice to local conditions.

## Background

Island colonization dynamics are of general interest in evolutionary biology, both with respect to understanding adaptive radiations, as well as for tracing migration patterns. In this context it is of particular interest to ask whether a single colonization can already result in a new established population that is refractory to further invasions, or whether multiple independent invaders are required before a new stable population can be established. This question can be particularly well studied in cases of recent island colonization, since this provides insights into the early phases of establishment and adaptation in a population context. The spread of the house mouse (*Mus musculus *L.) across many oceanic islands in contemporary times constitutes an excellent model system in this respect [1,2].

*Mus musculus *originated on the Indian subcontinent within the past million years and there are currently at least three recognized subspecies: *M. m. domesticus, M. m. musculus, M. m. castaneus *[3]. *M. m. domesticus *invaded Western Europe about 3,000 years ago [4] and then colonized the rest of the world (i.e. Africa, America and Australia/New Zealand) mostly in the wake of increased human travel across the globe that started in the 16^th ^century [1,3,4]. They were also very successful in colonizing isolated islands, such as those of the Southern Ocean [5-9], where they were brought by whaling ships making stops during their journeys or went for seal hunting.

**Figure 1:**
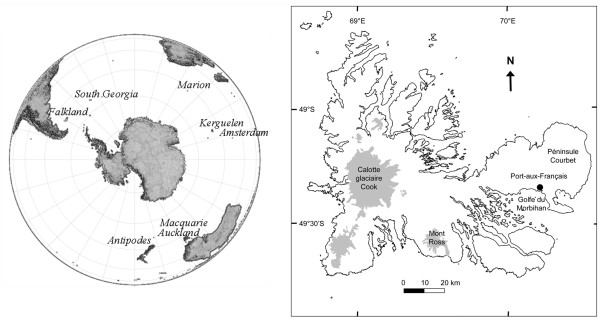
**Locations of the Southern Ocean islands assessed in this study (left) and map of the Kerguelen Archipelago (right)**. The sampling sites in the Kerguelen Archipelago are all around the Morbihan Gulf and the research station at Port-aux-Français except Port-Couvreux, Pointe du Morne and the Cap Ratmanoff (see further details in Figure 5).

The Kerguelen archipelago was discovered on the 12^th ^of February 1772 by Yves-Joseph de Kerguelen-Trémarec. It is situated about 4,000 km away from the African and the Australia coast (Figure 1a), has a large main island of 6,500 km^2 ^called Grande Terre, and approximately 60 small islands (1-200 km^2^) surrounding it (Figure 1b). The climate is Oceanic cold, characterized by cold summers (8°C on average), no rigorous winters (2°C on average), by strong wind and mean annual rainfall of 747 mm (350-1479 mm during 1951-2009, Météo-France, Port-aux-Français). There was never an extended human settlement on the archipelago, but since 1951, there is a research and weather station with a continuous turnover of about 60 to 120 people per year.

The house mouse was most likely introduced to the Kerguelen at the beginning of the 19^th ^century [10-12], but certainly not before 1772, since it is too far away from the continents to have been a destination for ship traffic in previous times. During the high times of whale and seal hunting, there was heavy boat traffic in this area, with a large potential to bring additional mice. Today, the mice have colonized all of Grande Terre as well as many of the small islands of the Morbihan Gulf [13,14].

With this defined history, as well as extensive data on the genetic diversity of the relevant source populations (Western Europe), we have an excellent test case to study population genetic consequences of island invasion, the subsequent spread to further islands and patterns of re-invasion.

## Results

### Mitochondrial Data

**Figure 2:**
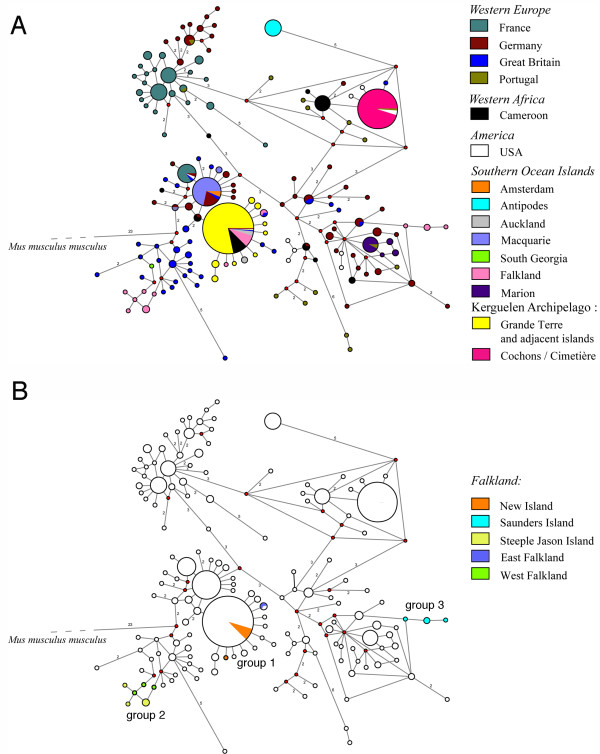
**D-loop haplotype networks calculated using Median Joining for *M. m. domesticus *samples with *M. m. musculus *as outgroup**. The size of the circles represents the frequency of the respective haplotype in our sample. Each node is one mutational step away from the next node, numbers indicate the cases where more than one step is required to join the nodes. Small red circles indicate branch splits. (A) General network including all published sequences that are related to the Kerguelen haplotypes. (B) Same network as in (A), but only with the Falkland samples highlighted.

We sequenced 834 bp of the mitochondrial control region (D-loop) from all samples and found that all haplotypes grouped within the known *M. m. domesticus *haplotypes (Figure 2a), i.e. belong to this subspecies. This was already known for some of the islands [8,9] and we show here that it is also the case for the Kerguelen Archipelago, Amsterdam Island, Falkland Islands and South Georgia. Hence, the source populations of the mice colonizing the small Southern Ocean islands came most likely from Western Europe, or via Atlantic Islands and North America which were colonized by Western European mice.

For the Kerguelen Archipelago mice, we identified two major haplotypes. One is a very common one that was previously found in Western Europe, Cameroon and USA and occurs also on other Southern Ocean islands including the Falkland Islands and Auckland Islands. We find it on Grande Terre as well as adjacent islands (colored yellow in Figure 2a). The second major haplotype is very different from the first one and was previously found in Portugal and in the USA, but is also related to a haplotype known from Cameroon. Within the Kerguelen Archipelago, it is restricted to two small neighboring islands in the Morbihan Gulf, namely Cochons Island and Cimetière Island (colored dark pink in Figure 2a).

In addition to these major haplotypes, we identified eight new haplotypes in the Kerguelen Archipelago, which are only a single or two mutational steps apart from the first major Kerguelen haplotype. It is therefore likely that these have arisen on the Kerguelen Archipelago. A comparable pattern of one major haplotype with several single step derivatives is also known for the mice that have colonized Madeira [15].

**Figure 3:**
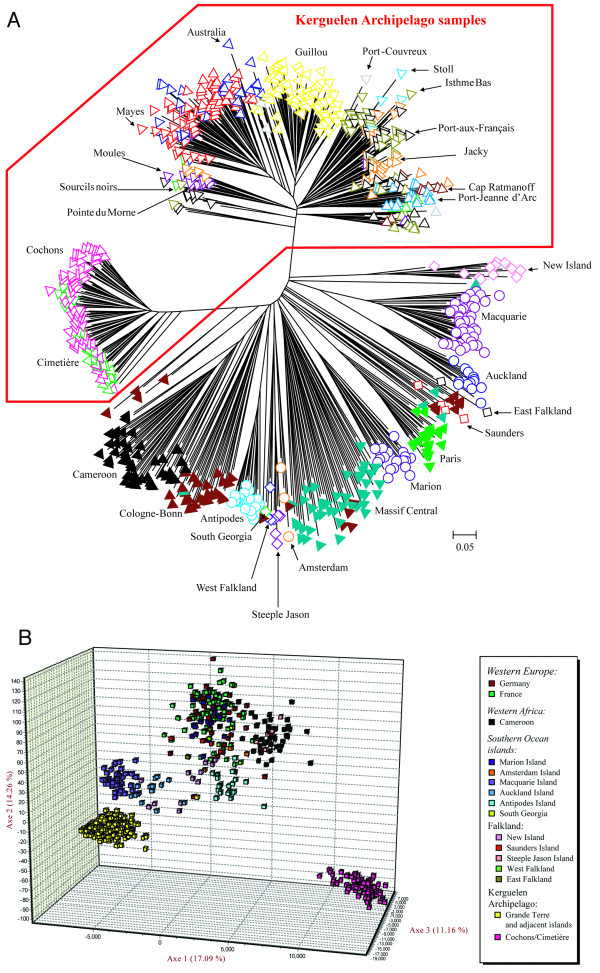
**Population structure based on autosomal microsatellite loci**. (A) Neighbor-joining tree based on the calculation of the proportion of shared alleles calculated for all individuals. Samples from the same location share the symbol/color pattern. (B) PCA plot with three axes displayed. Every square represents an individual, color patterns match the ones in (A).

To further assess whether this is a general pattern on small islands, we have more closely inspected the haplotype distribution on the Falkland Islands. We find three closely related haplotype groups (Figure 3b). The first group is identical to the Grand Terre haplotypes on the Kerguelen and occurs in East Falkland, as well as on New Island, which is in the far West of the archipelago. The second is related to haplotypes known from Great Britain and occurs on Steeple Jason Island, which lies far out in the North-West, as well as on West Falkland. The third is related to haplotypes known from Germany and Great Britain and is found on Saunders Island, which is very close to the Northern part of West Falkland. None of these islands has disparate sets of haplotypes, thus confirming the notion of single primary colonizations. On the other hand, the colonization pattern and history across the archipelago is apparently complex, since geographic proximity within the archipelago does not correlate with haplotype similarities. More intensive sampling will be required to unravel this further.

### Microsatellite data

A total of 18 unlinked autosomal microsatellites were typed for all samples and heterozygosities as well as average number of alleles were calculated for each sampling location (Table 1). All island samples show reduced heterozygosities (0.43 on average) and lower average allele numbers (2.9 on average) when compared to the standard samples from the European mainland populations Cologne-Bonn, Germany (0.84/11.7) and Massif Central, France (0.86/12.1). Such a reduced heterozygosity and lower allele numbers on the islands is in principle in line with the assumption of a colonization bottleneck, but the situation is more complex. It is known that local inbreeding and communal nesting occurs in natural populations of the house mouse [16], which can lead to local reduction of genetic diversity [17]. The sampling scheme for the German and French standard populations took account of this effect and took samples from an extended area to obtain a measure of the average genetic diversity in the extended area [17]. But the sampling on the islands could not be done in this way, since many are actually smaller than the areas considered for the standard sampling protocol. To compare the island results with an equivalent sampling scheme on the mainland, we typed samples that were all caught in the garden of the National Library of France in Paris. Average heterozygosity (0.47) and average allele number (3.0) is indeed also lowered for these and thus more comparable to the island samples.

**Table 1:** Population genetic parameters for microsatellite loci and mitochondrial sequences

		18 autosomal microsatellite loci				D-loop sequences	
area	location	N	H_exp_	H_obs_	A_av_	N	N_haplo_
*Main land populations*							
Germany	Schleswig-Holstein	-	-	-	-	9	8
	Cologne - Bonn	43	0.84	0.61	11.7	44	26
France	Paris	20	0.46	0.47	3.0	18	1
	Massif Central	46	0.86	0.75	12.1	62	19
Cameroon	Kumba	46	0.61	0.48	6.7	58	8
							
*Kerguelen archipelago*							
Grande Terre	Port-aux-Français	41	0.48	0.44	4.1	28	3
	Jacky	29	0.48	0.49	3.3	22	3
	Isthme Bas	38	0.48	0.46	3.9	38	1
	Cap Ratmanoff	8	0.46	0.49	2.7	6	1
	Pointe du Morne	1	0.28	0.56	1.6	1	1
	Port-Couvreux	4	0.47	0.49	2.6	4	2
	Port-Jeanne d'Arc	16	0.42	0.42	3.3	15	3
	Sourcils Noirs	5	0.38	0.43	2.3	5	1
Golfe du Morbihan	Moules Island	12	0.33	0.37	2.4	10	1
	Stoll Island	4	0.38	0.48	2.1	4	1
	Australia Island	28	0.43	0.38	3.5	27	1
	Mayes Island	71	0.41	0.36	4.0	71	2
	Guillou Island	79	0.36	0.34	2.4	79	1
	Cochons Island	69	0.36	0.35	2.4	65	1
	Cimetière Island	28	0.38	0.37	2.5	27	1
							
*Falklands*							
	New Island	12	0.44	0.41	3.2	12	2
	Steeple Jason	5	0.33	0.33	2.3	5	3
	Saunders Island	4	0.55	0.44	3.0	4	1
	East Falkland	2	0.49	0.64	2.3	2	1
	West Island	3	0.48	0.49	2.7	3	2
							
*other Southern Ocean islands*							
	Marion Island	18	0.56	0.51	4.3	18	2
	Amsterdam Island	3	0.49	0.54	2.6	3	1
	South Georgia	1	0.16	0.31	1.3	1	1
	Antipodes Island	18	0.44	0.51	3.1	17	1
	Macquarie Island	40	0.42	0.39	3.3	38	3
	Auckland Island	13	0.42	0.39	3.2	13	2

N = number of inviduals analysed, H_exp _= expected heterozygosity, H_obs _= observed heterzygosity, A_av _= is average number of alleles across loci, N_haplo _= number of different haplotypes found

Previous studies found evidence that there could be differences between male and female mediated gene flow patterns [15,18]. We have therefore also typed six Y-chromosomal microsatellites for those island samples where eight or more males were available (Table 2). For five of the Y-chromosomal loci we find only one major allele at most locations on the Kerguelen Archipelago, suggesting that only one Y-chromosomal haplotype has been involved in the colonization (Table 2). Only Y24 on Cimetière Island is fixed for a different allele, but this is a secondary effect, since these mice are derived from Cochons Island (see below) that harbor this allele at low frequency. Additional alleles are also found at other loci, but most of these are only a single mutational step away from the major allele and have thus likely been generated after the colonization. This explains also the diversity of alleles at locus Y22, since this locus appears to be generally hypervariable, suggesting a particularly high mutation rate. Interestingly, even this hypervariable locus has only a single major allele on Cochons Island and Cimetière Island, indicative of very recent colonization or strong bottleneck effects.

**Table 2:** Distribution of Y-chromosomal microsatellite allele frequencies

		Kerguelen Archipelago islands										Cameroon	other subantarctic islands		
locus	allele	Port aux Francais (28)	Jacky (13)	Isthme Bas (29)	Port Jeanne d'Arc (11)	Moules (8)	Australia (15)	Mayes (52)	Guillou (38)	Cochon (36)	Cimetière (16)	Kumba (21)	Marion (8)	Antipodes (12)	Maquarie (21)
Y6	120	1	1	1	1	1	1	1	1	1	1	0.95		1	
	122												0.75		1
	124											0.05	0.25		

Y12	118						0.07	0.02							
	124	1	1	1	1	1	0.93	0.96	1	1	1	0.93			
	129							0.02						1	
	132											0.07			
	135														0.1
	137												1		0.9
	140														

Y21	295											0.05	0.75		
	316	1	1	1	0.9	0.5	0.93	1	1	1	1	0.95			
	318				0.1	0.5	0.07								
	320													0.25	0.95
	321												0.25		0.05
	322													0.67	
	324													0.08	

Y22	239													0.67	
	253												0.25		
	255														
	257							0.1							0.05
	259						0.07	0.1				0.05			
	261											0.85	0.13		0.62
	263										0.06	0.1	0.5	0.25	0.24
	265	0.04					0.07	0.02		0.97	0.94		0.13		0.09
	267							0.02		0.03					
	269	0.04	0.38	0.1			0.4	0.35							
	271	0.18	0.15	0.03			0.13	0.04	0.16					0.08	
	273	0.4	0.15	0.14	1		0.2	0.25	0.84						
	275	0.21	0.23	0.14		0.14	0.13	0.08							
	277	0.11	0.08	0.31		0.57		0.02							
	279			0.24		0.29									
	281	0.04													
	291			0.03											
	294			0.03											

Y23	309											0.95			
	315					0.5									
	317														1
	319									0.03				1	
	321	0.93	0.01	0.93	1	0.5	1	1	1	0.97	1				
	323	0.07		0.07											
	325											0.05			
	329												1		

Y24	373											0.05	0.71		
	392												0.29		
	393	0.86	0.92	1	1	1	1	0.92	1	0.8		0.95			
	395	0.14	0.08					0.04		0.2	1				
	397							0.04						1	
	399														0.95

The Y-chromosomal allele patterns from the other islands that were typed (Marion Island, Antipodes Island and Macquarie Island) are very distinct from the ones that we found in the Kerguelen, with almost no overlap in the major alleles. Thus, they represent distinct Y-chromosomal haplotypes. All of the loci considered here were previously typed for the German and French populations and also showed a high diversity of alleles there (not shown). Intriguingly, however, for four of the loci the major alleles found in the Cameroon population correspond to the major alleles in the Kerguelen (Table 2). This suggests that there is some relationship of the Kerguelen mice to the Cameroon mice, albeit not necessarily a direct one, since the Cameroon population represents a new colonization by itself.

### Population relationships

To assess the population structure and relationships on the basis of the 18 autosomal microsatellites, we produced an allele sharing tree and run a PCA analysis (Figure 3). In the allele sharing tree, we find a coherent assignment of most populations and samples to distinct clades (Figure 3a). The sole exceptions are population samples from the Cologne-Bonn and Massif Central areas that are represented in multiple clades, likely reflecting their high diversity. With additional markers their genetic clustering was readily recovered in a previous study [17].

Two major clades are apparent within the Kerguelen Archipelago. The Cochons/Cimetière island samples are very different from all the other islands, although they appear to be somewhat associated to the Cameroon/German clade. Among the other islands, the Guillou Island samples form a single distinct clade and the island pair Australia/Mayes a separate mixed clade (Figure 3a). The Grande Terre samples as well as Moules Island and Stoll Island are mixed among each other, without clear distinction.

The PCA analysis is largely congruent with the allele sharing tree, but shows a stronger distinction of the two Kerguelen groups and no particular association of the Cochons/Cimetière island samples with the Cameroon/German clade (Figure 3b). On the other hand, it provides less resolution within each of the groups.

**Figure 4:**
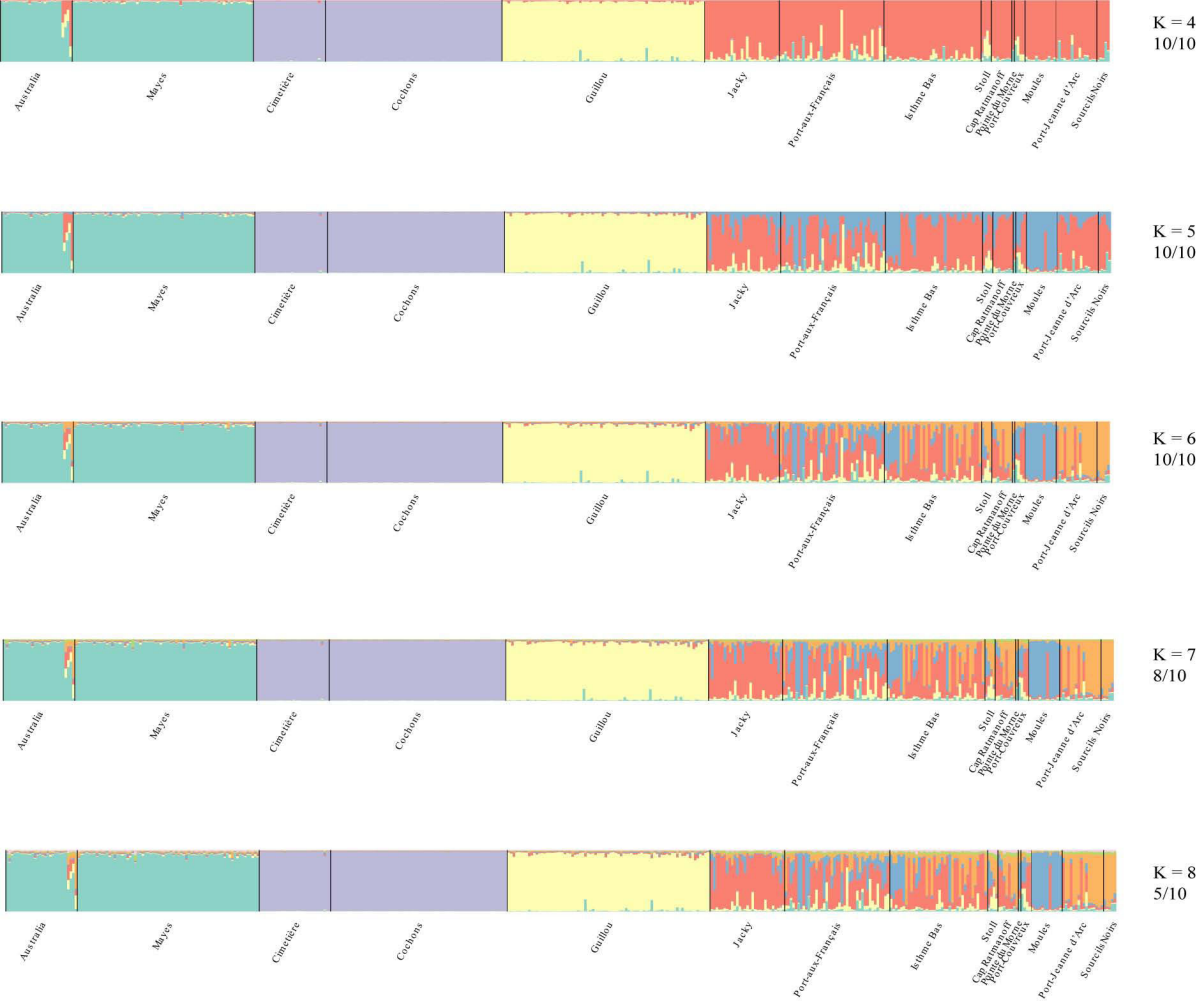
**Structure analysis within the Kerguelen Archipelago**. Only the results for the hypothesis of between 4 - 8 population groups (*K *= 4 to *K *= 8) are shown, represented by different colors. Each vertical bar represents a single individual, as well as its likelihood to belong to a given population group. The numbers below the *K *values represent the number of times that the same pattern was obtained in 10 independent runs of the program.

To study the population structure within the Kerguelen Archipelago further, we conducted an individual-based cluster analysis with the program Instruct [19]. To assess the possible number of clusters *K*, we performed runs with increasing numbers of *K *and recorded the likelihoods. A plateau was reached for *K *= 10 to 15, depending on the run, but the assignment of individuals to clusters was very unstable for these values, indicating that the lower *K *values reflect the true structure better. In Figure 4 we plot therefore only the results for values of *K *ranging from 4 to 8, alongside the number of runs that gave consistent assignments to clusters. We find that a value of *K *= 6 appears to be stable and we therefore use this for evaluation.

The structure analysis results thus confirm the pattern seen on the allele sharing tree. The island pairs Australia/Mayes and Cochons/Cimetière each form a single cluster and Guillou Island forms a clear separate cluster. The remaining locations are much more intermixed, only Moules Island is relatively homogeneous, although Moules-like genotypes appear to occur also in other locations. Interestingly, Stoll Island is mixed into Grande Terre populations but given the geographical location and its proximity to the main island (about 20 m), the mice might have originated from there (Figure 4).

**Figure 5:**
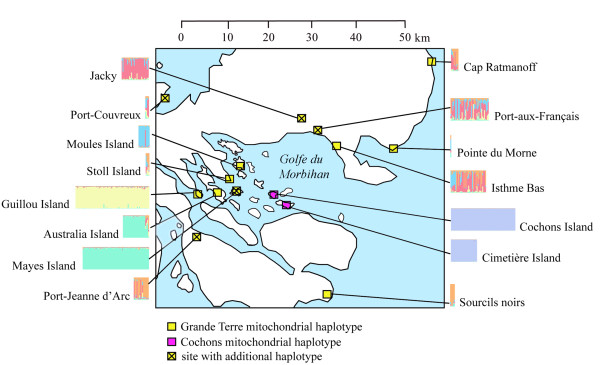
**Summary of population structure analysis and mitochondrial haplotype distributions across the Kerguelen Archipelago**.

Figure 5 provides a summary diagram showing the genetic structure of all sampling sites on the Kerguelen Archipelago. Note the clear distinction of the two mitochondrial haplotype groups and the clear structure results for Guillou Island and Moules Island, as well as the island pairs Mayes/Australia and Cochons/Cimetière.

## Discussion

### Primary colonization

Our data are compatible with the notion of an initial colonization of the Kerguelen Archipelago by a small group of mice, at the minimum the genetic equivalent of two females and one male. This can be inferred from the presence of two major mitochondrial haplotypes, as well as a single major Y-chromosomal haplotype. However, the distinct placement of the Cochons/Cimetière island samples in the allele sharing tree and the PCA analysis, as well as the presence of a single mitochondrial halotype only (i.e. no single step derivatives), suggests that these mice are in fact derived from a second more recent colonization event. Intriguingly, however, there is a similarity of the Y-chromosomal haplotypes of these mice with the rest of the Kerguelen mice. This seems unlikely to have occurred by chance, since the loci we have typed are generally polymorphic in the Western European mice. The three other island samples in our study (Macquarie Island, Antipodes Island and Marion Island) have indeed different major Y-chromosomal haplotypes (Table 2). Given that the Y-chromosomal haplotype from Cameroon is closely related to the Kerguelen haplotype, one could propose that both, the first and the second colonization came from Cameroon, which was itself colonized from Western Europe. The fact that both major mitochondrial haplotypes in the Kerguelen Archipelago are also identical or closely related to haplotypes found in Cameroon supports this notion. However, we are not confident that such a direct connection exists. Although historical ship journeys are known to have stopped both at Cameroon and the Kerguelen Archipelago, for example the German scientific expedition "Deutsche Tiefsee" in 1898 from La Valdivia [20], these usually have had several additional stops on other islands and it is very difficult to trace how many boats went on to the Kerguelen Archipelago and the routes they took. Thus, it seems also possible that other Atlantic islands or Atlantic harbors of the USA, where most whaling boats that went to the Kerguelen Archipelago came from (the first whaling expedition known came from Nantucket Island (USA) in 1792 - [21]), share the allele patterns with the Cameroon population and could thus have been the source population for a secondary invasion on Cochons/Cimetière Islands. Again we note that the two major mitochondrial haplotypes found in the Kerguelen occur also in the USA. More intensive sampling of the USA locations needs to be done before this question can be answered in a satisfactory way. Still, it remains noteworthy, and also unexpected, that two separate primary invasions on the Kerguelen have come from related source populations.

Although our study is focused on the Kerguelen Archipelago, we also identified interesting patterns for mice on the Falklands Islands. Both in the mitochondrial haplotype analysis, as well as in the allele sharing tree, different locations in the archipelago can be molecularly differentiated, and therefore may be regarded as different populations. Three mitochondrial haplotype groups were detected, whereby two of them are shared between disparate islands. Interestingly, West Falkland and Steeple Jason Island, which are about 40 km away from each other, share not only the mitochondrial haplotypes but are also grouped together in the allele sharing tree (Figure 3a). On the other hand, New Island and East Island, which share also the mitochondrial haplotypes, are very different in the allele sharing tree. The Falkland Islands have been regularly visited by boats from different nations (e.g. England, Spain, France etc.) and even today a population of around 3,000 Falkland Islanders lives there. Thus, the geographical location (near South America) and the presence of an extended human population should have increased the number of potential colonization events in the Falklands Islands. Still, it appears from our limited data that the different colonizations that have occurred on different islands of the archipelago may also have been resilient to re-invasions.

### Subsequent spread

As expected, the population and allele patterns found within the Kerguelen Archipelago allow some general conclusions on the fate of populations after initial colonization. First of all, we note that these mice have retained a certain amount of genetic diversity. The heterozygosity values, as well as the average number of alleles, are comparable to the sample that we caught within a single deme in Europe (Paris). Since the mice that came with the first ship would likely represent the deme from the harbor where the ship started, we can assume that the mice entering these ships had a similarly reduced diversity (when compared to the diversity across demes in the French and German populations). Hence, there may have been only little additional loss of genetic diversity during the ship passage and after colonization. In population genetic terms this means that the mouse population would have quickly expanded after arrival on the Kerguelen Archipelago, which would have prevented further loss of genetic diversity due to drift in small populations. Mice generally go through successions of population expansions and contractions between seasons every year (for Guillou Island, see [22]) suggesting that their life history patterns are well compatible with such a scenario.

There were further colonization cycles within the Kerguelen Archipelago, namely the ones that lead to the colonization of the islands in the Morhiban Gulf. The islands Guillou, Mayes and Australia are close to Grande Terre (< 500 m) and initial colonization might have occurred by animals that drifted there, or were transported by humans. Active swimming, as it was directly observed for rats [23] can also not be excluded, but seems less likely for small rodents due to the low water temperatures (about 5°C in summer). Nevertheless, many small islands close to Grand Terre harbor mouse colonies. The initial colonizers on the small islands would have quickly expanded and retained much of their genetic variation, although the allelic patterns are sufficiently distinct to make them genetically separable from the Grande Terre population. The Grande Terre samples, on the other hand, are not genetically distinct from each other, suggesting that they are connected by continuous gene flow. This shows at the same time that very little re-invasion of the smaller islands appears to occur, since their genetic distinctness appears to be maintained (i.e. not subjected to the high levels of gene flow that occur on Grande Terre).

In contrast to the islands close to Grand Terre, Cochons Island and Cimetière Island are located further away in the Morbihan Gulf and these are the ones where we indeed see a different pattern, namely a secondary invasion by mice not coming from Grand Terre (see above). They harbor only a single mitochondrial haplotype with no additional mutational variants (Figure 2) and also only a single major allele at the hypervariable Y-chromosomal locus Y22 (Table 2). This implies that the colonization has occurred later than that of the rest of the Archipelago. Indeed for Cimetière Island mice have only been recorded from 2002 onwards and it is possible that they were inadvertently transferred from the neighboring Cochons Island by humans. Another possibility could be a natural migration, since the distance between the islands is only tenths of meters a low tide. There is evidence to suggest that the island was frequently visited for whaling and fishing activities around 100 years ago when the first mice could have arrived, although cauldrons used for extracting fat from penguin can be found only on Cochons Island today. On Cochons Island the mouse population was also inadvertently affected by a rabbit eradication program using poison from 1992 to 1997 [24]. This could have resulted in a bottleneck and could thus explain the lowered genetic diversity. The same eradication program was also conducted on Guillou Island and could be the reason for low genetic diversity on this island as well as the different cluster in the structure and the allele sharing tree compared to other Kerguelen samples.

Apart from the Cochons/Cimetière Islands case, we have no evidence for secondary successful colonization across the entire Kerguelen Archipelago, although new mice must have frequently arrived every year during whaling times. In other island mice colonization studies, it was found that although mitochondrial patterns similarly suggest only a single invasion, there could still be continued male mediated gene flow [15,18]. However, given that we have only one major Y-chromosomal haplotype throughout the archipelago, this seems unlikely for the Kerguelen Archipelago. Hence, we can conclude that it must be difficult for newly arriving mice to invade the already occupied territory in the Kerguelen. Thus, our findings of single primary invasions and resilience to re-invasions corroborate the studies by Searle et al. [4,9,25], which have suggested that the phylogeographic patterns seen for mouse populations reflect ancient human movements, with only little disturbance by later movements. The successful experimental introduction of house mice into the Scottish Isle of May [26] does not contradict this conclusion, since in this case the mice came from another Scottish island with similar ecology, i.e. are expected to have had the same environmental adaptations at the time where they arrived.

### D-loop mutation rate

We identified several new mitochondrial haplotypes, mainly in Kerguelen, but also on the Falklands, Marion Island and Macquarie Island, most of which are only one step away from the major resident haplotype (Figure 2). These can be expected to have arisen only after colonization of the respective islands. We can therefore estimate a mutation rate based on the colonization time of approximately 200 years ago. A single mutation among 834 bp is equivalent to 0.12% sequence divergence which, when divided by 200 years, gives a mutation rate of 6 × 10^-6 ^per year. This is a factor of 150 higher than the estimate of 4 × 10^-8 ^per year for the intraspecific mutation rates of the same D-loop region suggested by [27,28], which is already higher than the interspecific rate. The dependence of such estimates on the coalescence times considered is a well known pattern in various taxa [29], although the reasons for this a still disputed [30,31]. The sequencing of the mitochondrial genomes of laboratory derived strains that were established about 100 years ago indeed suggests a 10-15 times higher mitochondrial mutation rate among such recently derived lineages, although no new mutations were found in the D-loop region [32]. But even taking this rate into account, our estimate is still a factor of 10 times higher, suggesting that another process must play a role. This could be selective sweeps caused by advantageous mutations elsewhere in the mitochondrial genome and providing a new adaptation in the respective matriline. For humans it has been suggested that such mutations do indeed occur and have specifically been fixed in individuals of populations living at higher latitudes indicative of providing an adaptation to the colder climates [33].

### Adaptation and genetic isolation

The ecological situation of the mice on the Kerguelen Archipelago is very different from Western European conditions, both with respect to the cold climate, as well as food conditions and the virtual absence of human settlements. Still, mouse densities can become very high, at least in regions where they have no predators [34]. Also, it has been shown for mice on sub-Antarctic islands that they have changed their preferred diet from plant seeds to macroinvertebrates for most of the year [14,35]. All of this indicates that mice are likely to be locally adapted to these conditions. This could explain why it is so difficult for newly arriving mice to invade the existing populations. They would not only have problems to become integrated into the existing social structure, but would also have to compete with better adapted competitors. Alternatively, this may be a simple statistical effect, given that newly arriving mice would usually be few in numbers and resident mice form a large population. Thus, even if newly arriving mice mate successfully with the resident mice, the new alleles and haplotypes that they carry might not rise to sufficient frequencies to make an impact on the overall pattern. On the other hand, given that the single colonization pattern appears to be consistently found on all small islands, it seems more likely that local adaptation plays a role as well. Interestingly, the colonization of the much larger and ecologically diverse New Zealand Island is characterized by multiple invasions, including different sub-species [9]. Thus, it seems possible that the mouse populations on small islands can become more quickly ecologically and genetically isolated than mouse populations on larger islands and thereby have a higher propensity to eventually form a new subspecies or species, possibly enhanced through the fast formation of new chromosomal races [36].

## Conclusions

Our data suggest that on small islands, the primary mouse colonization wave is decisive for the population that becomes established. Further introduction of mice from ships do not appear to have a big impact on the genetic composition of the resident population.

## Methods

### Mouse samples

Population samples from Cologne-Bonn (Germany), Massif Central (France) and Cameroon were described previously [17]. For these we had applied a sampling scheme that took care to sample the genetic variation within an area of about 50 km diameter (i.e. trapping sites were at least 300 meters away from each other). Hence we consider these samples to reflect the local population diversity. Additional samples from Schleswig-Holstein (Northern Germany) were trapped in 2006 using the same scheme. In contrast, the mice from Paris (n = 20) were caught within the confinements of the garden of the National French Library (BNF) in 2009, i.e. not following the extended sampling scheme above. The mice in the BNF are living in a space of around 1 ha at the center of the national library building. They are separated from other populations outside the BNF by poisoning. Hence, these are considered to represent a single sample from a local population, not necessarily reflecting the diversity in the extended area.

The Kerguelen Archipelago samples were caught mainly in the Morbihan Gulf area including several islands and the adjacent Grande Terre (see Table 1 for details). Again it was not possible to apply the extended sampling scheme in this case. Instead, the sampling followed the scheme described in [24]. All the mice in the Kerguelen Archipelago were captured in non-inhabited area except around the research station in Port-aux-Français. The mice were trapped using a line system, with three parallel lines 40 m away from each other and a length of approx. 100 m each with 34 traps along the line (1 trap every 3 m). Mice from Port-aux-Français (n = 41), Guillou Island (n = 79), Cochons Island (n = 69), Isthme Bas (n = 38), Mayes Island (n = 18), La cabane dite "Jacky" (n = 29), Cimetière Island (n = 28), Australia Island (n = 24), Port-Jeanne d'Arc (n = 16), Cap Ratmanoff (n = 8), Sourcils noirs (n = 5), Port-Couvreux (n = 4) and Pointe du Morne (n = 1) were trapped in 2008 and 2009. Mice from Moules Island (n = 12) and Stoll Island (n = 4) were captured in 2005. Other Mayes Island (n = 57) and Australia Island (n = 4) mice were trapped in 1996.

Amsterdam Island (n = 3) samples were collected in December 2007. Marion Island mice (n = 18) were caught at two localities across the island, namely at the Meteorological Station and at Mixed Pickel Cove in 1990 (n = 6) and 2004 (n = 12) [8]. Macquarie Island (n = 12), Antipodes Island (n = 18) and Auckland Islands (n = 13) mice where caught in 2005-2006 [9]. Additional samples from Macquarie Island (n = 28) from 2005 were used. Falkland Islands samples from New Island (n = 12) were caught in 2006 [37] and 2010 (n = 18). Samples from the other Falkland Islands namely Saunders Island (n = 4), Steeple Jason Island (n = 5), East Falkland (n = 2) and West Falkland (n = 3) and a mouse from South Georgia were caught in 2008/2009.

### D-loop sequencing

DNA was extracted using salt extraction. The D-loop was amplified using the primers 5'-CATTACTCTGGTCTTGTAAACC and 5'-GCCAGGACCAAACCTTTGTGT. The reactions were carried out in 10 μL final volume with the following cycling parameters: 95°C for 15 minutes followed by 35 cycles of 95°C for 30 s, 60°C for 1.30 min, 72°C for 1 min and 15 min at 70°C for elongation time. Exo-Sap purification (USB Corp.) was performed with the following incubation: 37°C for 20 min and 80°C for 20 min. The cycle sequencing reaction parameters were 96°C for 1 min followed by 29 cycles of 96°C for 10 s, 55°C for 15 s and 60°C for 4 min.

The sequences generated were visualized using CodonCodeAligner Ver. 2.0.1 (CodonCode Corp.) BioEdit ver.7.0.9.0 [38] and MEGA ver. 4 [39]. The haplotype data file was calculated using DnaSP 4.50.3 [40]. The network was calculated using the Median Joining method and drawn with Network ver. 4.5.1.0 (Fluxus Technology Ltd), taking care that missing data did not affect the network [41]. The sequences were submitted to Genbank and are available under accession numbers HQ185258 to HQ185282.

### Microsatellite typing

From a previously described set of 1,000 microsatellites [42,43], we chose 18 (Chr01_25, Chr02_01, Chr03_21, Chr03_24, Chr04_31, Chr05_15, Chr05_45, Chr07_38, Chr08_11, Chr09_20, Chr11_64, Chr12_05, Chr13_22, Chr14_16, Chr16_21, Chr17_09, Chr18_08, Chr19_08) which were known to be polymorphic in the German and French populations. Six Y-chromosomal loci which we found to be polymorphic in the German and French populations were also typed for all island samples where more than 8 males were available. Primer sequences used to type the Y-chromosome were: Y6 aaccaccactatcttcattc and acagagtatacgtacgtgtg, Y12 cccaatctaggcatttaatt and attcaccattctccagtgtg, Y21 accatcagatgatcaccaagtgc and tccagcattcaatggtacaggct, Y22 tcatggtagacaccatggcaac and tcagttttctaggtggaggggtg, Y23 acctcactcaggatgatgccctc and agcctgtgcgcacgtgtgtg, Y24 tctgggggtttcgggtggagcct and gcatcacagctgaggctctgtgg. Forward primers were labeled with FAM or HEX dye on the 5' end. The reactions were carried out in 5 μL final volumes using 10 ng DNA template using a multiplex PCR kit (Qiagen). The PCR conditions were: 95°C for 15 min followed by 28 cycles at 95°C for 30 s, 60°C for 1.30 min, 72°C for 1.30 min with a final extension at 72°C for 10 min. PCR products were diluted 1:20 in water. 1 μL of this dilution was added to 10 μL of HiDi formamide and 0.1 μL of 500 ROX size standard (Applied Biosystems). The denaturation step was performed with the following incubation times: 90°C for 2 min and 20°C for 5 min. The alleles were analyzed using GeneMapper ver. 4.0 software (Applied Bioscience). The distances for the allele sharing tree were calculated using MSA3.15 [44]. The tree was generated using R and drawn using MEGA4 [39]. Structure was analyzed using Instruct [19] because this method does not assume Hardy Weinberg equilibrium within loci. The run parameters were as follow: 2 chain number, a burn-in period of 100,000 simulations followed by a run length of 2,000,000 MCMC simulations and ten iterations for each *K *(number of clusters). To draw the structure diagram the softwares CLUMPP (version 1.1.2 [45]) and Distruct [46] were used. The PCA was generated using the software Genetix 4.03 [47].

## Authors' contributions

EH developed the project, did the laboratory work and wrote the first draft of the manuscript. JLC organized the sample collection on the Kerguelen and on the National French Library (Paris), MS, JC, BV and PQ provided D-loop sequences, DNA and further samples from the other islands, RS and MT developed the Y-chromosomal primers and provided the information on allele patterns in Cameroon and Europe. DT was the primary supervisor and participated in its design and coordination and wrote the final manuscript. All authors were involved in writing and data interpretation, and read and approved the final manuscript.
